# Functional Dynamics of Deafferented Early Visual Cortex in Glaucoma

**DOI:** 10.3389/fnins.2021.653632

**Published:** 2021-07-26

**Authors:** Gokulraj T. Prabhakaran, Khaldoon O. Al-Nosairy, Claus Tempelmann, Markus Wagner, Hagen Thieme, Michael B. Hoffmann

**Affiliations:** ^1^Department of Ophthalmology, Otto-von-Guericke University, Magdeburg, Germany; ^2^Department of Neurology, Otto-von-Guericke University, Magdeburg, Germany; ^3^Center for Behavioral Brain Sciences, Otto-von-Guericke University, Magdeburg, Germany

**Keywords:** fMRI, glaucoma, lesion projection zone, plasticity, visual cortex, feedback

## Abstract

In advanced retinitis pigmentosa with retinal lesions, the lesion projection zone (LPZ) in the early visual cortex can be driven during visual tasks, while it remains unresponsive during passive viewing. We tested whether this finding translates to advanced glaucoma, a major cause of acquired blindness. During visual stimulation, 3T fMRI scans were acquired for participants with advanced glaucoma (*n* = 4; age range: 51–72) and compared to two reference groups, i.e., advanced retinitis pigmentosa (*n* = 3; age range: 46–78) and age-matched healthy controls with simulated defects (*n* = 7). The participants viewed grating patterns drifting in 8 directions (12 s) alternating with uniform gray (12 s), either during passive viewing (PV), i.e., central fixation, or during a one-back task (OBT), i.e., reports of succeeding identical motion directions. As another reference, a fixation-dot task condition was included. Only in glaucoma and retinitis pigmentosa but not in controls, fMRI-responses in the lesion projection zone (LPZ) of V1 shifted from negative for PV to positive for OBT (*p* = 0.024 and *p* = 0.012, respectively). In glaucoma, these effects also reached significance in V3 (*p* = 0.006), while in V2 there was a non-significant trend (*p* = 0.069). The general absence of positive responses in the LPZ during PV underscores the lack of early visual cortex bottom-up plasticity for acquired visual field defects in humans. Trends in our exploratory analysis suggesting the task-dependent LPZ responses to be inversely related to visual field loss, indicate the benefit of patient stratification strategies in future studies with greater sample sizes. We conclude that top-down mechanisms associated with task-elicited demands rather than visual cortex remapping appear to shape LPZ responses not only in retinitis pigmentosa, but also in glaucoma. These insights are of critical importance for the development of schemes for treatment and rehabilitation in glaucoma and beyond.

## Introduction

Glaucoma, a progressive degeneration of retinal ganglion cells (RGCs), results in an irreversible loss of vision, eventually leading to blindness (Jonas et al., 2017). Worldwide it is the second most prevalent cause of acquired blindness, next to cataract (Quigley and Broman, 2006). Most of the conventional therapeutic strategies, by medication or surgery, are directed toward the management or control of the major risk factor in glaucoma, increased intra-ocular pressure (IOP). In fact, these interventions are known to reduce the progression rate of the disease (Jonas et al., 2017). However, the beneficial effects of recent advances in early detection and progression-delaying treatment of glaucoma are counteracted by increased life expectancy. As a consequence, a substantial proportion of glaucoma patients will become severely visually impaired and eventually bilaterally blind during their lifetime (Quigley and Broman, 2006; Kapetanakis et al., 2016). This motivates current research initiatives, not only to focus on better disease treatment tools, but also to further our understanding of the management of visual impairment in advanced glaucoma. In fact, the ultimate goal is to explore effective avenues for the restoration of visual input, which is motivated by ongoing research progress, ranging from cell-based therapies at the retinal level to interventions at the cortical level (reviewed in Jutley et al., 2017; Roska and Sahel, 2018). For this purpose, knowledge about the state and functionality of the deafferented visual cortex in glaucoma is instrumental. In fact, in addition to the retinal damage caused by glaucoma, the concomitant deprivation of visual input from the retina to the cortex has been shown to result in structural and functional changes at the cortical level, in particular in the primary (V1) and extra-striate (V2 and V3) visual cortex (Duncan et al., 2007; Dai et al., 2013; Frezzotti et al., 2014; Boucard et al., 2016; Wang et al., 2016; Zhou et al., 2017).

fMRI-based retinotopic mapping demonstrated the interplay of plasticity and stability in congenital visual pathway abnormalities and inherited retinal diseases (Hoffmann et al., 2012; Hoffmann and Dumoulin, 2015; Ferreira et al., 2017; Ahmadi et al., 2019, 2020). In contrast, reorganization of the primary visual cortex is much more limited in patients with acquired visual field defects as studied in detail for macular degeneration (Baker et al., 2008; Masuda et al., 2008; Dilks et al., 2009; Liu et al., 2010; Baseler et al., 2011; Plank et al., 2013). Although glaucoma is a prevalent disease, the scope of cortical plasticity in the deafferented portions of the early visual cortex in advanced glaucoma has only received little attention. Zhou et al. (2017) reported an enlarged para-foveal representation in the visual cortex of glaucoma patients, but recent investigations of simulated peripheral response dropouts in controls (Prabhakaran et al., 2020) suggest that such effects do not necessarily reflect veridical long-term cortical reorganization. This mirrors the views of previous reports analyzing the limited nature of visual cortex plasticity for foveal de-afferentation (Baseler et al., 2011; Haak et al., 2012; Barton and Brewer, 2015). Accordingly, reductions of amplitude and extent of fMRI BOLD responses in the visual cortex of glaucoma patients (Duncan et al., 2007; Song et al., 2012; Borges et al., 2015; Murphy et al., 2016) and thinning of gray matter in the de-afferented visual cortex (Yu et al., 2014, 2015; Boucard et al., 2016) suggest the absence of large-scale reorganization post visual field loss.

Importantly, in the context of vision restoration and rehabilitation strategies, investigations of the responsivity of lesion projection zones (LPZ) in the visual cortex, i.e., cortical representations of the retinal lesions, are key to understanding the reality of adult visual cortex reorganization capabilities. Consequently, investigations are needed to assess cortical responses in the LPZ and their relation to visual stimulation and visual tasks. In fact, for patients with non-glaucomatous retinal disorders, cortical activations in the LPZ were reported. Remarkably they appear to depend on the presence of a visual task to be performed on the presented stimuli. In a series of case observations Masuda et al., demonstrated these task-dependent V1-responses in patients with macular degeneration (MD) and retinitis pigmentosa (RP), i.e., for central (Masuda et al., 2008, 2020) and peripheral visual field defects (Masuda et al., 2010), respectively. These task-dependent effects have now been confirmed in a larger cohort of RP patients (*n* = 13) using spatially specific stimulation (Ferreira et al., 2019). These signals are taken as evidence for an absence of bottom-up plasticity and are discussed as side effects of task-related feed-back and attentional demands from higher visual areas. Although highly relevant for the management of advanced glaucoma, such insights into the responsiveness of the LPZ in the early visual cortex are currently completely missing for the entity of glaucoma patients.

In the present study, we assessed the task-dependence of cortical responses in the LPZ in a set of glaucoma patients (GL), carefully selected to have extensive glaucoma-related peripheral visual field defects, and compared the findings to those in two reference groups, i.e., advanced RP and controls with simulated peripheral visual field defects. In fact, LPZ response signatures and task-dependencies in RP and glaucoma matched and were analogous to those reported previously in MD, but absent in controls. Consequently, the lack of relevant bottom-up plasticity appears to be a general feature of the human visual system.

## Materials and Methods

### Participants

Demographics of the participants is given in Table 1. Participants with extensive visual field (VF) defects due to advanced open-angle glaucoma (GL; *n* = 4) or to retinitis pigmentosa (RP; *n* = 3; RP2 also had secondary glaucoma, see Table 1) and age-matched visually healthy controls (HC) with normal vision [best-corrected decimal visual acuity ≥ 1.0 (Bach, 1996); *n* = 7] took part in the study. Written informed consents and data usage agreements were signed by all participants. The study was conducted in adherence to the tenets of the Declaration of Helsinki and was approved by the ethics committee of the University of Magdeburg.

**TABLE 1:** Participant demographics and clinical characteristics.

**Participants**	**Age**	**Sex**	**Stimulated eye (fMRI)**	**Visual acuity**	**Visual field (MD—dB)**	**Fixation stability^1^ (2°)**	**Onset age (yrs)**	**Duration (yrs)**
GL1	68	Female	Left	1.0	−13.9	100%	59	9
GL2	70	Male	Left	0.4	−25.8	100%	65	5
GL3	51	Female	Right	0.8	−32.5	na	48	3
GL4	72	Male	Left	0.8	−30.7	100%	47	25
RP1	56	Female	Left	0.6	−23.5	97%	26	30
RP2^2^	78	Male	Left	0.05	−30.7	99%	59	19
RP3	46	Female	Right	0.05	−27.3	31%	11	34
HC1	64	Male	Left	1.6	1.0	na	na	na
HC2	79	Female	Left	1.3	0.0	na	na	na
HC3	56	Male	Left	1.3	0.0	na	na	na
HC4	73	Male	Left	1.0	−0.9	na	na	na
HC5	75	Male	Left	1.3	0.0	na	na	na
HC6	71	Female	Right	1.0	−1.4	na	na	na
HC7	63	Female	Left	1.0	na	na	na	na

*GL, glaucoma; RP, retinitis pigmentosa; HC, control participants; MD, Visual field mean deviation as measured with standard automated perimetry (SAP); na, not available.*

*^1^Proportion of central fixations within a fixation window of 2° radius as determined with a fundus-controlled perimeter (see section “Materials and Methods.”).*

*^2^RP participant with secondary glaucoma; visual field defects are predominantly driven by the primary disease, i.e., RP, consequently, RP2 is classified as RP.*

### Visual Field Testing and Fixation Stability

Standard automated perimetry (SAP) was performed using 24–2 Swedish Interactive Threshold Algorithm protocol (SITA-Fast; Goldmann size III white-on-white stimuli; Humphrey Field Analyzer 3; Carl Zeiss Meditec AG; Jena, Germany). For three participants, VF to the central 30° was tested using another perimeter (OCTOPUS^®^ Perimeter 101, Haag-Streit International, Switzerland; dG2; dynamic strategy; Goldmann size III). For the patient cohort (except 1; GL3), fixation stability was determined with a fundus-controlled microperimeter (MP-1 microperimeter, Nidek, Padua, Italy) during fixation of a central fixation target. Fixations were tracked with 25 Hz and the proportion of fixations falling within the central 2° radius was determined using built-in MP1 analysis (Table 1).

### Visual Stimulation for fMRI

#### Stimulus Conditions and Rationale

Three different tasks were performed independently in separate runs within the same session: (1) one-back task (OBT), (2) passive viewing (PV), and (3) fixation-dot task (FDT). The underlying rationale was to dissociate top-down modulations and bottom-up input to the visual cortex by applying visual stimulation with a moving pattern with (OBT) and without a stimulus related task (PV). Specifically, during (1) OBT, the participants were instructed to report a repetition of same drifting directions of the pattern in two consecutive trials using a button press. They were required to fixate on the central dot while performing the task. One-back repetition trials were ensured to be at least 15% of the total number of trials and were randomized. All the participants were able to perform the stimulus-locked task without much difficulty. (2) During PV, the participants passively viewed the stimulus while fixating the central dot, i.e., they were explicitly instructed not to do the OBT during the PV. (3) The FDT task was introduced as a reference condition to test whether the effects of PV could be enhanced by fixing the participants’ attention at the center to make it more difficult to perform a self-paced stimulus related task, e.g., the OBT. For FDT (not locked to the stimulus, i.e., running during both on- and off-blocks) the participants responded via button press when the color of the fixation dot changed. In all controls and most of the patients, the switch-colors used were black and white; however, in some patients different colors were used depending on the ability of participants to notice the change. The color change occurred throughout the cycle i.e., during both the stimulus presentation and the mean luminance gray. The spatial and temporal properties of the stimuli were kept consistent for all the three conditions. Each of the different task-condition was repeated for three times in an interleaved order (ABCCBAABC; A-PV, B-OBT, C-FDT) and the sequence was kept the same for all participants. Before each run, the participants were informed of the current task by the MRI technician through an audio setup in the scanner.

#### Visual Stimulation

**FIGURE 1:**
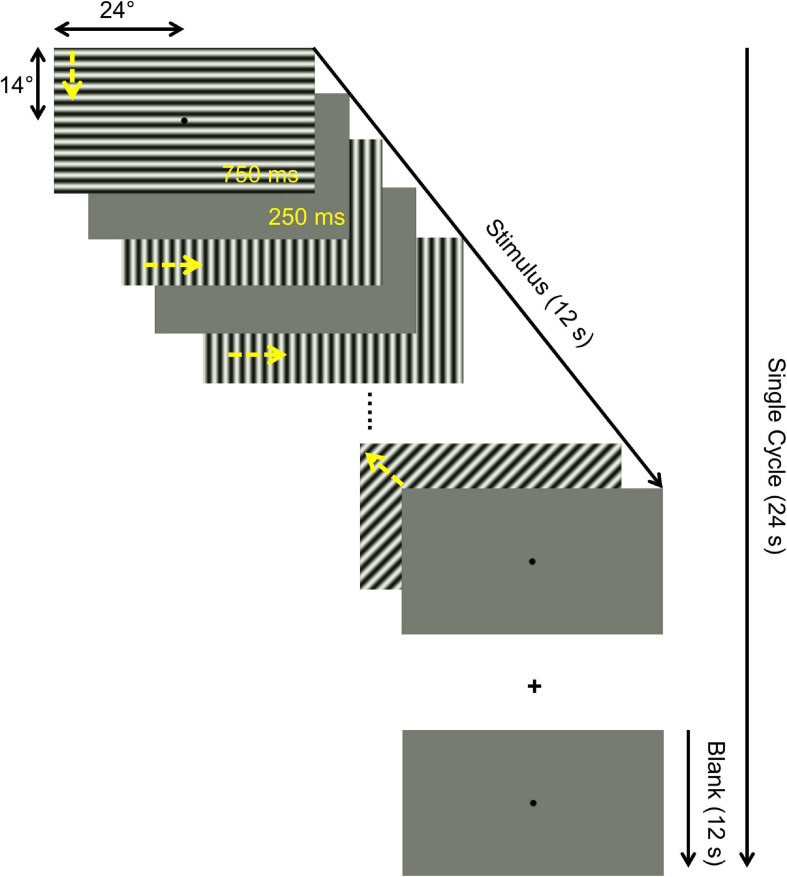
Illustration of temporal sequence of a single stimulus cycle. Each 24 s cycle comprised a 12 s motion block (drifting patterns) block and a 12 s mean blank block (mean luminance gray). A total of 10 cycles were presented per fMRI run, resulting in a total duration of 240 s. The motion block consisted of 12 1-s trials comprising 750 ms motion stimulus (drifting in one of 8 different directions, as indicated by yellow arrows added for visualization) and 250 ms mean luminance gray. Identical stimuli were presented for three different task conditions in separate fMRI runs: (i) passive viewing (PV), (ii) one-back task (OBT), (iii) fixation-dot task (FDT), as detailed in section “Materials and Methods”.

Psychtoolbox-3 (Brainard, 1997; Pelli, 1997) was used to program the visual stimuli in MATLAB (Mathworks, Natick, Massachusetts, United States). The stimulus employed comprised high-contrast patterns drifting in eight different directions that were projected to a screen at the rear end of the magnet bore, with a resolution of 1,920 × 1,080 pixels. Participants viewed the stimulus monocularly via the better eye [patients: based on SAP (MD and extent of VF-loss); controls: dominant eye] at a distance of 35 cm via an angled mirror. This resulted in an effective stimulus size subtending approximately 24 and 14° radius in the horizontal and the vertical directions, respectively. All the patients viewed the stimulus projected on the entire screen, whereas, in the controls, we simulated an artificial peripheral scotoma by exposing only the central 7° of the stimulus through a circular aperture. The temporal sequence of each run followed a block design with 10 cycles of 12 s motion block (stimulus presentation) alternating with 12 s of mean luminance gray (24 s per cycle). Within each motion block, the direction of the contrast pattern was randomly changed every second (i.e., 12 trials per block; Figure 1). In each 1 s trial, the stimulus was presented for 750 ms followed by a 250 ms mean luminance gray. Participants were instructed to maintain fixation on a centrally located fixation dot. The stimulated eye of the participants were monitored and evaluated via an eye tracker qualitatively during fMRI measurements. The spatial properties of the stimulus are quite robust to account for any possible eye-movement related influence in the fMRI response. In particular, even in patients with little eye-movements, the representation of the scotoma and intact VF remains the same because of the presence of the stimulus on the entire screen.

In addition, to delineate the visual areas, fMRI-based population receptive field (pRF) mapping scans were obtained from each participant in a second session on a separate day. A checkerboard stimulus pattern (mean luminance: 109 cd/m^2^; contrast: 99%; check size: 1.57°) moving in eight different directions (2 horizontal, 2 vertical and 4 diagonal; Dumoulin and Wandell, 2008) was exposed through a bar aperture. The width of the bar subtended 1/4th (3.45°) of the stimulus radius (13.8°). The spatial and temporal properties of the stimulus have been described in Prabhakaran et al. (Prabhakaran et al., 2020). The duration of each pRF mapping scan was 192 s and the scan was repeated 6 times for the patient cohort and 4 times for the controls. The participants responded to a fixation-dot color change via button press.

### MRI Acquisition

All MRI and fMRI data were collected on a 3 Tesla Siemens Prisma scanner (Erlangen, Germany). In order to allow for an unrestricted view of the entire projection screen, we used only the lower section of a 64-channel head coil, resulting in a 34-channel coil covering most of the brain. fMRI scans parallel to the AC-PC line were acquired using a T2^∗^-weighted BOLD gradient-EPI sequence (TR | TE = 1,500 | 30 ms and voxel size = 2.5^3^ mm^3^). A total of 160 fMRI time series images (volumes) were obtained for each run after the removal of the first 8 volumes by the scanner itself to allow for steady magnetization. The fMRI parameters were the same for the pRF mapping data, except for the number of volumes (136). One high-resolution whole brain anatomical T1-weighted scan (MPRAGE, 1 mm isotropic voxels, TR | TI | TE = 2,500 | 1,100 | 2.82 ms) was collected for each participant to allow for cortical visualization of fMRI responses. An inversion recovery EPI sequence (TR | TI | TE = 4,000 | 1,100 | 23 ms) with spatial coverage (FOV) and resolution identical to the T2^∗^ EPI was obtained to aid in the alignment of structural and functional data.

### Data Preprocessing

Gray-white matter boundaries in the T1-weighted anatomical images were segmented using Freesurfer^1^. ITK-gray was used to manually inspect the Freesurfer segmentation and correct for possible segmentation errors^2^. A 3-D rendering of the cortical surface was generated by reconstruction of the segmented boundaries (Wandell et al., 2000). Within and between fMRI scans, head motion artifacts were corrected using AFNI^3^. For each participant, motion-corrected fMRI time series of the repetitions of each stimulation condition (i.e., PV, OBT, FDT, and pRF mapping data) were averaged into separate groups with MATLAB-based Vistasoft tools (mrVista^4^). The inversion recovery EPI was aligned spatially with the anatomical scan in two steps; first manually with rxAlign function in mrVista and then automatically using Kendrick Kay’s alignment toolbox^5^. The obtained alignment matrix was used to align the fMRI images with the anatomy.

### Data Analysis

#### Phase-Specified Coherence (Coherence_*ps*_)

We computed voxel-wise coherence at the fundamental stimulus frequency and phase corrected for hemodynamic delay (phase-specified coherence) to quantitatively investigate changes in the strength of the BOLD response for the different task conditions. We used the definition and formula to calculate the phase-specified coherence (coherence*_*ps*_*) that has been previously used (Masuda et al., 2008) and is also available as a function in the mrVista toolbox.

$${\text{Coherence}}_{ps}=\frac{A_{0}}{\sqrt{\Sigma A_{f}^{2}}}\text{cos}\left(\phi_{0}-\varphi \right)$$

where A_0_ and ϕ_0_ are the signals amplitude and phase at the stimulation frequency, respectively, A_*f*_ are the amplitudes of each Fourier component, and φ is the delay in the hemodynamic response (estimated for each participant from the positive fMRI responses). The phase at the stimulus frequency was estimated from the averaged fMRI time-series of all the task conditions in a small 5 mm ROI drawn in the region of the cortex that had reliable positive BOLD response across all the conditions. Coherence_*ps*_ can take values between −1 and +1; voxels with positive measure reflect stimulus synchronized fMRI response modulation and negative measures reflect modulation to the mean luminance gray (no or negative stimulus related BOLD response).

#### Visual Area Delineation

We defined the borders of primary (V1) and extra-striate visual cortex (V2 and V3) for each participant using fMRI-based pRF-mapping data. Employing a 2D-Gaussian pRF model approach described previously (Dumoulin and Wandell, 2008; Prabhakaran et al., 2020), we estimated for each voxel their preferred position in the visual field (x and y in Cartesian coordinates). Eccentricity $\sqrt{(x^{2}+y^{2}}$) and polar angle $\tan^{-1}\left(\frac{y}{x}\right)$ measures were derived from these position parameters. Polar angle estimates were projected onto an inflated cortical surface and the visual areas were delineated by following the phase reversals in the polar angle data (Sereno et al., 1995). As in advanced glaucoma and RP, retinal and subsequent cortical lesions render this delineation process difficult, due to deafferentation and hence disrupted maps, it was complemented by visual area definitions based on the Benson atlas (Benson et al., 2014). The Bensons atlas applies for each individual an anatomically defined template of retinotopy and thus informs pRF-mapping based visual area definitions. The anterior extent of the visual areas was manually drawn based on the participants pRF mapping data and Benson atlas extracted eccentricity predictions (14° in the vertical meridian representation and 24° in the horizontal meridian representation) in correspondence to our stimulus size. Based on the coherence*_*ps*_* measures from passive viewing (PV), we divided each visual area into two ROIs; voxels with positive responses were classified as the normal projection zone (NPZ) and those with negative responses as the lesion projection zone (LPZ).

All further region of interest (ROI) analyses were performed with custom written scripts in MATLAB and statistics in SPSS 26 (Statistical Package for the Social Sciences, IBM). For each visual area (V1, V2, and V3) and ROIs (LPZ and NPZ), separate two-way repeated measures ANOVAs were performed to test for the effects of *task* (PV, OBT, and FDT) and *group* (glaucoma, RP, control) on coherence*_*ps*_* and their significance, if any. Paired *t*-tests were used for *post hoc* comparisons and corrected for multiple comparison with the Holm-Bonferroni correction (Holm, 1979).

## Results

### Task-Dependent Responses in Lesion Projection Zone (LPZ)

**FIGURE 2:**
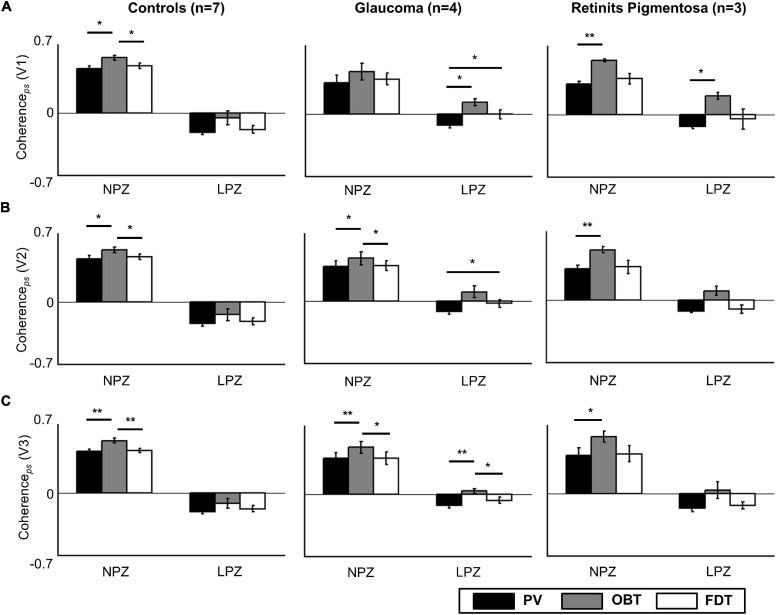
Task dependence of coherence*_*ps*_* in V1 **(A)**, V2 **(B)**, and V3 **(C)** for the control (*n* = 7), glaucoma (*n* = 4), and retinitis pigmentosa groups (*n* = 3; mean ± SEM). Controls and patients differ specifically for LPZ. Only in the patient groups’ LPZ, negative coherence*_*ps*_* during PV is shifted to positive values for OBT. Significance tests (corrected paired *t*-tests) on task effects were performed for all the participant groups, *p < 0.05 and **p < 0.01. PV, passive viewing; OBT, one-back task; FDT, fixation-dot task.

Firstly, as a validation and replication of the existing literature, we examined the scope of aberrant cortical responses in the deafferented visual cortex of a reference group of three participants with RP. Consistent with the results from Masuda and colleagues (Masuda et al., 2010) in their cohort of three RP participants primary visual cortex (V1), we found task-dependent activity in the peripheral LPZ in V1 (OBT-PV: *t* = 16.8; *p* = 0.012), and non-significant trends in V2 (OBT-PV: *t* = 6.2; *p* = 0.075) and V3 (OBT-PV: *t* = 3.7; *p* = 0.198) (see Figure 2, Supplementary Figure 1, and Supplementary Table 1).

**FIGURE 3:**
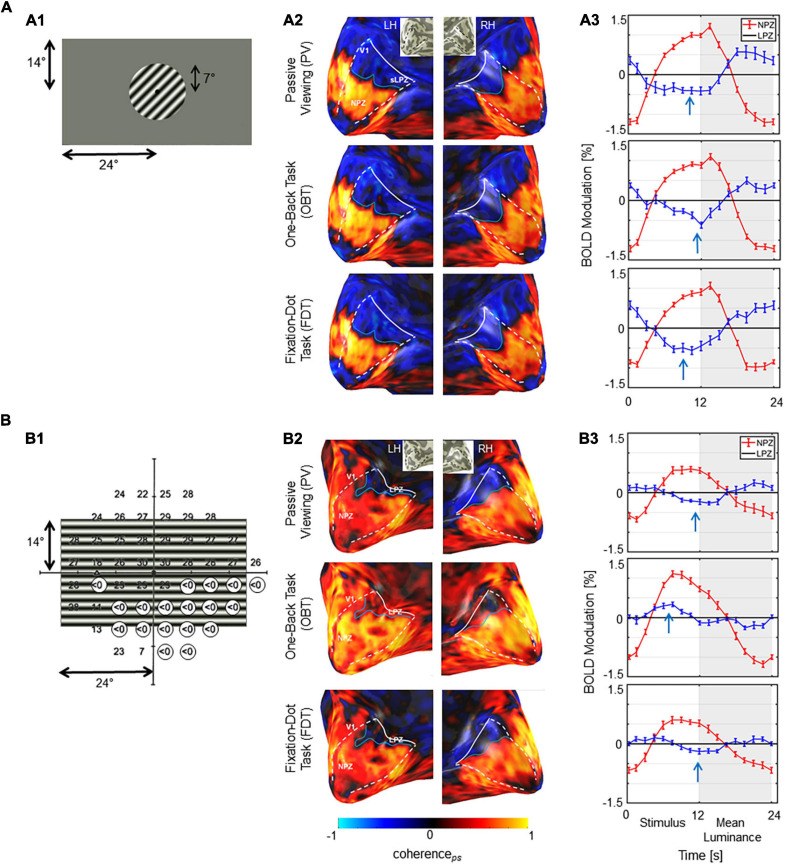
Stimulus schematics and fMRI-based activations. **(A)** Control HC1 **(A1)** Illustration of stimulated visual field for the controls with the simulation of a scotoma by peripheral masking beyond 7°. **(A2)** BOLD-activations (coherence_*ps*_) for the three task conditions projected onto the inflated occipital lobe as false-color overlays. V1 boundaries (white dashed) and the anterior extent (white solid line) were determined from the participants pRF mapping; NPZ and LPZ are indicated. **(A3)** Average single-cycle BOLD time series for the three conditions in the NPZ (red) and LPZ (blue) ROIs. White and gray zones indicate motion and blank blocks, respectively. The induced BOLD response is shifted due to the hemodynamic delay. Stimulus related BOLD modulations in LPZ are negative, irrespective of the task condition, as indicated by the arrows. **(B)** Glaucoma participant GL1. **(B1)** Visual field sensitivities for the study eye (left eye SAP) as determined perimetrically are superimposed onto stimulus layout (absolute scotomas are highlighted by white discs). **(B2)** BOLD-activations (coherence_*ps*_) depicted as for A2. V1 was determined from the participants pRF mapping data informed by atlas mapping as detailed in section “Materials and Methods.”. **(B3)** Average single-cycle BOLD time series depicted as for A3. Depending on task, LPZ responses were negative (PV/FDT) or positive (OBT), as indicated by the arrows. NPZ, normal projection zone; LPZ, lesion projection zone; RH, right hemisphere; LH, left hemisphere.

Next, we explored task-dependent LPZ responses in participants with advanced VF defects due to glaucoma. For a qualitative assessment, the fMRI-responses in the visual cortex of a representative participant with glaucoma (GL1) and a healthy control (HC1) with simulated peripheral VF-defect are shown in Figure 3 for three different stimulation conditions (PV, OBT, FDT). In the healthy control, LPZ responses were largely similar for all stimulation conditions, i.e., negative coherence*_*ps*_* and BOLD modulations. In contrast, LPZ responses in the glaucoma patient resulted in positive coherence*_*ps*_* and BOLD modulations for OBT, while negative coherence*_*ps*_* and negative BOLD modulations, as for the control, were obtained only for PV and FDT. Taken together, the response signatures for the participant with glaucoma resembled those reported for RP (Supplementary Figure 1).

**FIGURE 4:**
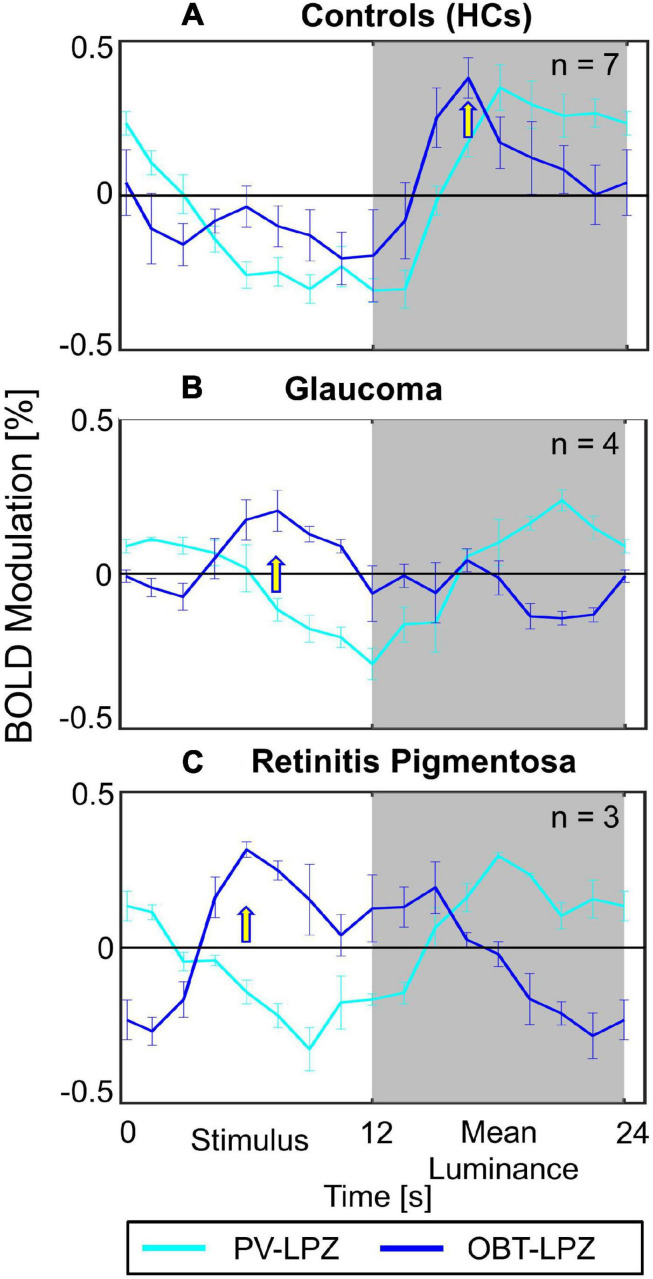
Average single-cycle BOLD time series ± SEM. Group-averaged fMRI response modulation across participants of V1 LPZ during PV (cyan) and OBT (blue) conditions. The panels show the plots for **(A)** HCs **(B)** Glaucoma **(C)** Retinits Pigmentosa. Yellow arrows indicate time-points with peak responses for OBT; the BOLD-peak for OBT shifts from the second block (12–24 s) in HC to the first block (first 12 s) in RP and glaucoma.

In a quantitative assessment, the above task dependence of the LPZ-responses in glaucoma was further confirmed at the group level. Each participant’s mean phase-specified coherence (coherence*_*ps*_*) was extracted from NPZ and LPZ voxels for each of the three stimulation conditions. The mean coherence*_*ps*_* of the three participant groups is depicted for V1 in Figure 2. For all participant groups, in NPZ we observed a strong positive coherence*_*ps*_*, which was enhanced for OBT. In contrast, in LPZ the negative coherence*_*ps*_* for PV turned positive for OBT in the glaucoma and RP group, while it remained negative, albeit reduced, for the control group. This differential effect between patient and control groups in LPZ was also directly evident from the inspection of the average single-cycle BOLD time series in LPZ. As depicted in Figure 4 for the group averages, only for glaucoma and RP did negative responses during stimulation (first 12 s) for PV shift to positive responses for OBT. As a consequence the BOLD-peak for OBT shifts from the second block (12–24 s) in healthy controls to the first block (first 12 s) in RP and glaucoma.

The significance of the task effect on V1 coherence*_*ps*_* and its difference across participant groups were assessed with two-way repeated measures ANOVA [between-subject factor: *group* (glaucoma, RP, control); within-subject factor: *task* (PV, OBT, FDT)] for the LPZ and NPZ separately. The effect of *task* was significant for both LPZ and NPZ (*p* < 0.001; see Supplementary Table 1), but the effect of *group* was significant only for the LPZ (*p* < 0.05). No significant interactions (*task* × *group*) were observed. *Post hoc* tests (corrected) were performed to identify the significant comparisons as indicated in Figure 2A. To assess the task-dependence of LPZ responses in glaucoma, the comparisons of the conditions OBT and PV in the control and glaucoma group are of particular relevance: for glaucoma there was a significant difference (*t* = 5.4; *p* = 0.024), but not for the controls. This effect was also significant in glaucoma V3 (*t* = 10.2; *p* = 0.006), while the same trend in V2 (*t* = 2.8; *p* = 0.069) failed to reach significance (Figures 2B,C). We further tested in glaucoma for any hierarchical effects of task-dependence in V1, V2, and V3 by calculating the effect size (computed as Cohen’s d) of the observed task-related coherence*_*ps*_* modulations in LPZ. We observed a stronger effect size in V1 (*d* = 0.70) and relatively lower effect size for V2 (*d* = 0.50) and V3 (*d* = 0.51). However, a repeated measures one-way ANOVA with *visual areas* (V1–V3) as the within-subject factor did not show any significant difference on the task-dependent effects across the visual areas [*F*_(2, 6)_ = 1.1; *p* = n.s.]. Finally, it was assessed whether the FDT-condition might serve as a better reference condition than PV by resulting in more negative coherence_*ps*_ values in LPZ. However, this was not the case, modulations for FDT fell short of that for PV, an effect that reached significance in glaucoma for V1 and V2.

### LPZ Responses as a Function of Clinical Characteristics

Considering that our patients (both glaucoma and RP) demonstrated significant task-dependent LPZ responses, we employed an exploratory approach to investigate the relationship of the magnitude of difference in coherence*_*ps*_* (i.e., OBT minus PV) with patient specific clinical characteristics. Specifically, the clinical measures included visual acuity (VA), disease duration, percentage of visual field loss (VF*_*loss*_*), mean deviation (MD), foveal sensitivity. VF*_*loss*_* was computed from the SAP-based VFs as the ratio of number of non-responsive test points vs. the total number of test points and considered only the regions of the VF representing our fMRI stimulus size. The relationship of the percentage of differential coherence*_*ps*_* in LPZ with these clinical measures was tested with a simple linear regression model [R^2^(adjusted), *p* < 0.05]. We did not find a significant association of the task-dependent responses with any of the afore-mentioned clinical correlates, except for MD and task-effect in the visual area V2. Nonetheless, based on the magnitude of R^2^, a negative trend in the task-effect with both MD (V1: 0.29; V2: 0.49) and the percentage of VF*_*loss*_* (V1: 0.18; V2: 0.38) in V1 and V2 was indicated, but not for V3.

### Cortical Thickness as a Measure of Structural Integrity

In an exploratory analysis, we probed for the presence of structural changes of the visual cortex following visual field deficits in patients compared to the controls. Freesurfer derived cortical thickness estimates, measured as the distance between the gray/white boundary and the pial surface (Fischl and Dale, 2000) was used as the indicator for structural integrity. For each participant and visual area (V1–V3) voxel-wise cortical thickness was extracted. Based on eccentricity estimates from a participant-specific anatomy driven retinotopic atlas (Benson et al., 2014), 12 ROIs of 2° eccentricity bins (0–24°) were created for each visual area and the mean cortical thickness was computed for each ROI. To investigate for differences in cortical thickness across groups a repeated measure ANOVA with *participant group* (patients and controls) as between-subject factor and *eccentricity* (0–24°) and *visual areas* (V1–V3) as within-subject factors was performed. The subject-specific global cortical thickness was included as a covariate to account for participant variability. While there was an overall trend for smaller cortical thickness in the *patient group* [cortical thickness controls vs. patients (mm) mean ± SEM: 1.76 ± 0.03 vs. 1.65 ± 0.02 (V1); 1.99 ± 0.05 vs. 1.93 ± 0.05 (V2); 2.31 ± 0.07 vs. 2.08 ± 0.05 (V3)], this did not reach significance. No other significant main effects or interaction were found. In addition, we tested for any potential association between the duration of visual field deficits and cortical thickness across the patients and did not find any significant relationship [*R*^2^: 0.08 (V1); 0.12 (V2); 0.20 (V3)].

## Discussion

We report that activations in the LPZ of the early visual cortex were strongly related to performing a one-back task in glaucoma and retinitis pigmentosa (RP), but not in controls with simulated LPZ. This indicates that the limited remapping, previously reported in RP and MD, is also a feature in glaucoma. These results thus suggest that strong limits of bottom-up plasticity are a general feature of the early human visual cortex that was de-afferented due to acquired lesions at the level of the retinal photoreceptors or ganglion cells.

The response pattern we observed, i.e., LPZ activation for visual stimulus-locked tasks, had previously not been reported for glaucoma. At the same time, it fits well into the context of other studies on visual plasticity in acquired retinal defects (for e.g., MD, Masuda et al., 2008, 2020; Baseler et al., 2011; RP, Masuda et al., 2010; Ferreira et al., 2019). From the initial apparent heterogeneity of reports on the scope of plasticity in the LPZ of human V1, ranging from the absence of relevant cortical remapping (Sunness et al., 2004; Baseler et al., 2011) to large-scale reorganization (Baker et al., 2008; Dilks et al., 2009), the picture of limited bottom-up plasticity in human V1 has eventually emerged (Wandell and Smirnakis, 2009; Morland, 2015). Accordingly, Masuda et al. (2008) observed LPZ-responses in V1 during the performance of stimulus-related visual tasks and concluded that they were associated with task-dependent feedback instead of bottom-up plasticity in MD (Masuda et al., 2008) and RP (Masuda et al., 2010). In a larger RP cohort, Ferreira et al. (2019) demonstrated similar task-dependent responses using spatial specific stimulation, as opposed to full-field stimulation employed by Masuda and colleagues (2020). This, in addition to recent findings of LPZ responsiveness to be modality independent [i.e., induced also by auditory and tactile stimulation associated tasks (Masuda et al., 2020)], adds further evidence to the hypothesis of LPZ responses to be dependent on the demands of task. In conclusion, we here demonstrate that these mechanisms are not specific to the rare disease RP, but they also apply to the much more prevalent disease entity of glaucoma.

### Early Visual Cortex Stability and Plasticity

The potential of remapping in V1 in acquired defects is often inferred from adult animal models (Kaas et al., 1990; Gilbert and Wiesel, 1992; Giannikopoulos and Eysel, 2006). While some degree of developmental V1 plasticity has been reported for congenital vision disorders (Baseler et al., 2002), the nature and magnitude of a large-scale reorganization in adulthood is questionable (Wandell and Smirnakis, 2009) and still warrants quantitative ascertainment. The differential comprehension in the above literature on LPZ activation primarily arises from the variable definitions of cortical reorganization and remapping (Morland, 2015). While the proponents of plasticity speculate that the mere presence of abnormal LPZ responses is sufficient evidence, the critiques point out the need for such responses to be non-explainable by the normal visual cortex organization following visual field loss. In the context of the latter definition of cortical reorganization, from our data, bottom-up large-scale reorganization appears an unlikely cause of the reported LPZ activation in glaucoma, as it would lead to LPZ responses irrespective of the condition, i.e., task. While this supports top-down effects as a cause of the task-related LPZ responses, these do not appear to strictly follow the inverse visual hierarchy, i.e., a decreasing effect size from V3 to V1, as might be expected for top-down modulations. In fact, the differential activation (reflected by Cohen’s d) we observed in V1 was not exceeded by those in V2 and V3 and did not reach statistical significance in a respective ANOVA. A stronger effect size in the extra-striate areas (i.e., V3 > V2 > V1) would have added further evidence to a top-down hypothesis. Further research is necessary to decipher the nature of the top-down modulations. One rewarding avenue to pursue for this purpose is paved by the advent of MRI at submillimeter resolution (Ress et al., 2007; Yakupov et al., 2017; Fracasso et al., 2018; Kashyap et al., 2018). It opens the possibility to recover information on the directionality of information flow in the cortex via laminar imaging (Dumoulin et al., 2018; Lawrence et al., 2019) that allows to dissociate activations in cortical input and output layers. Consequently, future studies measuring layer-specific functional activity in the visual cortex might unravel the missing pieces of information, i.e., origin and directionality of task-related LPZ activations, to validate or invalidate existing theories on the aberrant cortical activity observed in patients with de-afferented visual cortex.

### Clinical Relevance in the Context of Emerging Therapeutic Interventions

A subset of glaucoma patients continues advancing toward blindness regardless of disease management. While restoration therapies might offer treatment options for this patient entity, it has been suggested that vision loss associated changes at the level of visual cortex might be a reason for treatment failure in such cases (Gupta and Yücel, 2007; Davis et al., 2016; Nuzzi et al., 2018). Remarkably, the existence of cortical responses in the de-afferented visual cortex, as demonstrated in the present study, suggests that the LPZ is still to some degree operational. These finding are of particular importance for current promising initiatives to restore the visual input to the cortex (Roska and Sahel, 2018), which include retinal gene (Ashtari et al., 2015; Aguirre, 2017; Russell et al., 2017) and stem-cell therapy (Schwartz et al., 2015; Jutley et al., 2017; Mead et al., 2018; Wang et al., 2020), and retinal (da Cruz et al., 2016; Edwards et al., 2018) and visual cortex implants (Beauchamp et al., 2020). Our findings demonstrate the relevance of fMRI-investigations of the functionality of the visual cortex for the preparation of such demanding vision restoration procedures, e.g., via the individualized prediction of their clinical effectiveness.

### Relationship of Cortical Responses With Clinical Correlates

The presence and magnitude of LPZ task-dependent responses are likely also related to the patient’s clinical characteristics and their variability (Ferreira et al., 2019). Testing this, we observed a non-significant trend for the task-elicited LPZ responses to be negatively associated with the VF loss, i.e., the smaller the deficit the larger the response. This might suggest that the task-dependent LPZ-activations do not depend on the presence of extensive scotomas. Given the limited sample size in the present study, this explorative observation disserves following up in future studies. Nevertheless, the findings emphasize the benefits of patient stratification strategies for the investigation of disease related changes in the visual cortex.

### Limitations and Future Directions

As a primary limitation in the study, we acknowledge the small sample size of glaucoma patients, which was still fully sufficient to identify the relevant effects. It should be noted, that our target patients were those with strongly advanced VF defects. Consequently, as glaucoma is an age-progressive disorder, such patients are mostly in their later stages of life and likely to have at least one MRI-related contraindication, which makes them a rare cohort for fMRI investigations. Studies investigating the dynamics of the observed task-dependent LPZ responses in patients with different stages of the pathology are limited (Ferreira et al., 2017, 2019) which should be addressed by future research and more importantly aim to uncover the mechanisms underlying such responses with submillimeter laminar fMRI imaging.

Our definition of the lesion projection zone (LPZ) was based on negative modulations observed with full-field visual stimulation in accordance with previous studies (Masuda et al., 2010; 2008), but not on explicit spatial VF mapping. In consideration to previous studies (Plank et al., 2017) reporting changes in BOLD response modulation with stimulus type (for e.g., faces vs. checkerboard), it is to be noted that there might be a marginally differential characterization of LPZ with a full-field stimulus (in this case, gratings) compared to a spatial mapping stimulus. Importantly, in the context of population receptive field properties, distinctions in the cortical representation of visual field has been even reported for different spatial mapping-based approaches (Alvarez et al., 2015). As we tested for the presence of task-dependent responses in advanced glaucoma with substantial VF loss, full-field stimulation eliciting robust fMRI responses was preferred over a precise spatial delineation of the LPZ with spatially specific stimuli. Given the fact, irrespective of the stimulus type, keeping it consistent across the different conditions sufficed for our objective, the choice of stimulus could be rationalized. Nevertheless, a comparative analysis of LPZ definitions with different stimulation approaches might be of relevant interest to understand the dynamics of LPZ boundaries.

Some of our patients (GL2, RP2, and RP3) had quite low visual acuity and one of these patients (RP3) was predicted to have unreliable fixation with fundus-controlled perimetry, and the quantitative eye tracking data might have been informative. Despite this limitation, the qualitative monitoring and evaluation of the stimulated eye during scanning indicated all our participants to have good fixation of the stimulus in the scanner. In addition, given that the intact VF of our low visual acuity patients was largely constricted to central few degrees, we believe the responsive central visual field to be well within the stimulus window for potential small or moderate eye movements, thereby mitigating any possible eye-movement related influence in the fMRI response.

We also acknowledge a potential confound in our study with one RP patient also diagnosed with secondary glaucoma. Considering that, RP is not the primary disease under investigation in this study and used as a reference cohort to demonstrate the replicability and reproducibility of previous reports of task-dependent LPZ responses, we did not see a reason to exclude this patient in our analysis.

It should also be noted that our characterization of artificial scotoma was based on a generalized pattern of VF deficits as observed in peripheral vision disorders and not aiming at the simulation of our patient cohort’s idiosyncratic individual VF deficits. Although we acknowledge benefits of the latter approach, as our primary aim was to look at the task-dependent effects at a group level rather than individual patient-to-control comparison, the employed approach in the present study has the benefit of better within-group comparability. In addition, future research should also explore alternative methods to efficiently simulate artificial scotomas which closely correspond to patient like VF deficits, for e.g., via retinal bleaching (Magnussen et al., 2001; Gaffney et al., 2013) with high luminance levels and temporarily desensitizing the locations intended to represent an artificial scotoma.

## Conclusion

In summary, we demonstrated in patients with advanced glaucoma, the existence of aberrant cortical responses in the supposedly de-afferented regions of the early visual cortex. The fMRI modulations are more likely to be driven by task-elicited top-down neural mechanisms than bottom-up cortical reorganization. Given similar findings in glaucoma as in RP and MD, the results are indicative of a general mechanism behind such aberrant cortical responses that is not limited to the distinct pathophysiology of a specific disease. We believe that these insights are of importance for the development of treatment and rehabilitation schemes in glaucoma and beyond.

## Data Availability Statement

The raw data supporting the conclusions of this article will be made available by the authors, without undue reservation.

## Ethics Statement

The studies involving human participants were reviewed and approved by the Ethics Committee—University of Magdeburg. The patients/participants provided their written informed consent to participate in this study.

## Author Contributions

GP: conceptualization, methodology, formal analysis, investigation and data curation, and writing—original draft. MH: conceptualization, methodology, supervision, writing—review and editing, and funding acquisition. KA-N: investigation and writing—review and editing. CT: methodology and writing—review and editing. MW: investigation and writing—review and editing. HT: writing—review and editing. All authors contributed to the article and approved the submitted version.

## Conflict of Interest

The authors declare that the research was conducted in the absence of any commercial or financial relationships that could be construed as a potential conflict of interest.

## Publisher’s Note

All claims expressed in this article are solely those of the authors and do not necessarily represent those of their affiliated organizations, or those of the publisher, the editors and the reviewers. Any product that may be evaluated in this article, or claim that may be made by its manufacturer, is not guaranteed or endorsed by the publisher.

## Footnotes

1 https://surfer.nmr.mgh.harvard.edu/

2 https://github.com/vistalab/itkgray

3 https://afni.nimh.nih.gov/

4 https://github.com/vistalab/vistasoft

5 https://github.com/kendrickkay/alignvolumedata
