# Supplementary figures and images for: Functional Dynamics of Deafferented Early Visual Cortex in Glaucoma
Source: Front Neurosci. 2021 Jul 26;15:653632. doi: 10.3389/fnins.2021.653632 (PMC8350780; doi:10.3389/fnins.2021.653632)

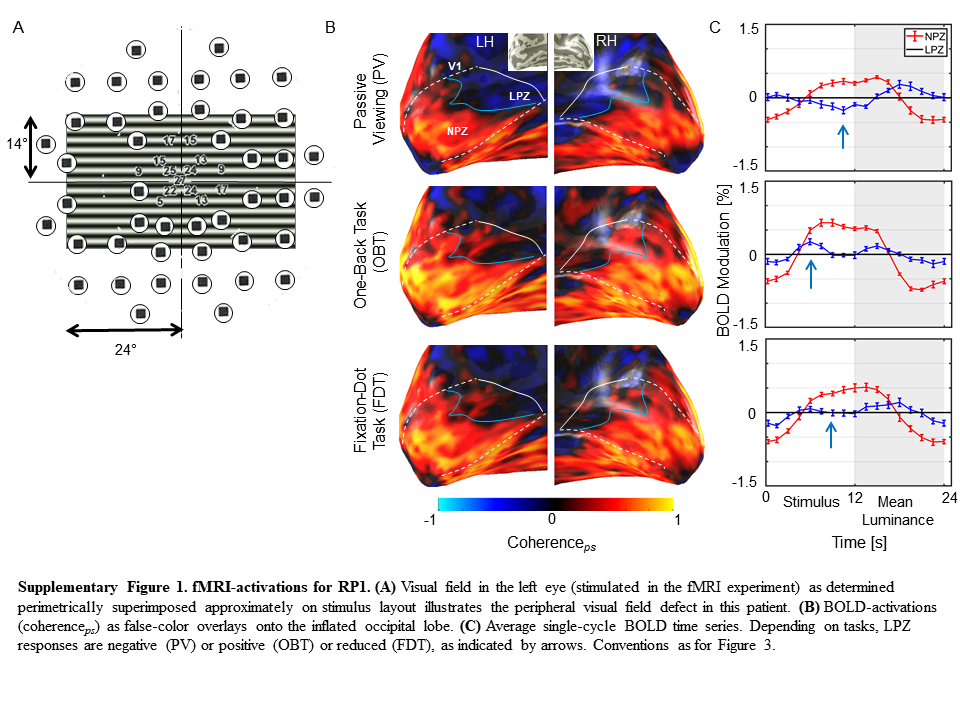

Supplement: Supplementary file 1 [file Image_1.tif]
