# Supplementary material for: Functional Dynamics of Deafferented Early Visual Cortex in Glaucoma
Source: Front Neurosci. 2021 Jul 26;15:653632. doi: 10.3389/fnins.2021.653632 (PMC8350780; doi:10.3389/fnins.2021.653632)
Supplement: Supplementary file 2 [file Table_1.docx]

**Supp Table S1.** Results of two-way repeated measures ANOVAs [between-subject factor: *group* (glaucoma, RP, control); within-subject factor: *task* (PV, OBT, FDT)] of the reported coherence*_ps_* measures for the LPZ and NPZ separately. Significant p-values are highlighted bold. Results of subsequent posthoc tests are indicated in Figure 3.

| **Visual Area** | **ROI** | **Main effect** | | | | **Interaction** | |
| --- | --- | --- | --- | --- | --- | --- | --- |
|  |  | ***Task*** | | ***Group*** | | ***Task x Group*** | |
|  |  | F (2,22) | p | F (2,11) | p | F (4,22) | p |
| V1 | LPZ | 26.3 | **< 0.001** | 4.1 | **0.048** | 1.4 | 0.267 |
|  | NPZ | 39.7 | **< 0.001** | 2.8 | 0.101 | 2.7 | 0.054 |
|  |  |  |  |  |  |  |  |
| V2 | LPZ | 12.5 | **< 0.001** | 8.3 | **0.006** | 1.0 | 0.426 |
|  | NPZ | 41.9 | **< 0.001** | 1.8 | 0.214 | 3.2 | **0.031** |
|  |  |  |  |  |  |  |  |
| V3 | LPZ | 19.3 | **< 0.001** | 2.9 | 0.097 | 1.3 | 0.308 |
|  | NPZ | 53.7 | **< 0.001** | 0.5 | 0.595 | 1.5 | 0.224 |
|  |  |  |  |  |  |  |  |
